# (*E*)-2-[2-(3-Nitro­phen­yl)ethen­yl]quinolin-8-ol

**DOI:** 10.1107/S1600536813027815

**Published:** 2013-10-16

**Authors:** Mathias Schulze, Wilhelm Seichter, Edwin Weber

**Affiliations:** a Institut für Organische Chemie, TU Bergakademie Freiberg, Leipziger Strasse 29, D-09596 Freiberg/Sachsen, Germany

## Abstract

In the title compound, C_17_H_12_N_2_O_3_, the mean planes of the benzene ring and the quinoline moiety are inclined to one another by 11.0 (1)°. The nitro substituent is twisted at an angle of 7.9 (2)° with respect to the attached benzene ring. Intra­molecular O—H⋯N and C—H⋯N hydrogen bonds occur. The crystal is constructed of mol­ecular stacks without involvement of π-stacking inter­actions, but showing inter­stack association *via* O—H⋯O and C—H⋯O hydrogen bonding. Thus, the supramolecular architecture of the crystal results from stacked molecules stabilized by hydrogen bonding between the stacks.

## Related literature

For uses of quinolin-8-ol and derivatives as complexants and pharmaceuticals, see: Albrecht *et al.* (2008 ▶); Cacciatore *et al.* (2013 ▶); Desvignes & Leguen (1963 ▶); McMaster & Bruner (1935 ▶); Vögtle & Weber (1979 ▶); Weber & Vögtle (1975 ▶). For applications of stilbene and derivatives, see: Butkovic *et al.* (2011 ▶); Ho *et al.* (2000 ▶); Navadiya *et al.* (2008 ▶); Ravikrishnan *et al.* (2012 ▶); Waibel *et al.* (2009 ▶); Zhu *et al.* (2013 ▶). For the preparative method used for the synthesis of the title compound, see: Yuan *et al.* (2012 ▶). For non-classical hydrogen bonds, see: Desiraju & Steiner (1999 ▶). For related structures, including intra­molecular hydrogen bonding of quinolin-8-ol, see: Faza­eli *et al.* (2008 ▶); Malecki *et al.* (2010 ▶); Yoneda *et al.* (2002 ▶); Zeng *et al.* (2007 ▶).
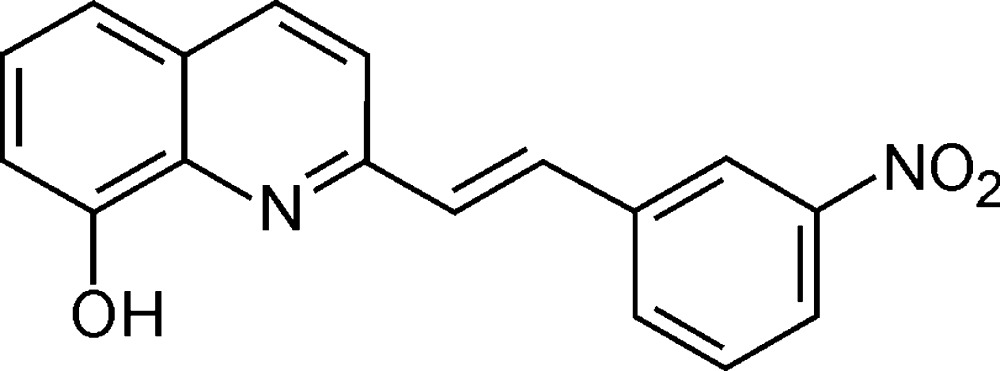


## Experimental

### 

#### Crystal data

C_17_H_12_N_2_O_3_
*M* *_r_* = 292.29
Monoclinic, 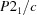

*a* = 20.3346 (7) Å
*b* = 4.7167 (1) Å
*c* = 15.5674 (6) Å
β = 109.255 (2)°
*V* = 1409.58 (8) Å^3^
*Z* = 4
Mo *K*α radiation
μ = 0.10 mm^−1^
*T* = 298 K
0.54 × 0.24 × 0.06 mm

#### Data collection

Bruker X8 APEXII CCD detector diffractometer
Absorption correction: multi-scan (*SADABS*; Bruker, 2007 ▶) *T*_min_ = 0.950, *T*_max_ = 0.994
22418 measured reflections
2655 independent reflections
1768 reflections with *I* > 2σ(*I*)
*R*_int_ = 0.028

#### Refinement

*R*[*F* ^2^ > 2σ(*F* ^2^)] = 0.065
*wR*(*F* ^2^) = 0.226
*S* = 1.03
2655 reflections
200 parameters
H-atom parameters constrained
Δρ_max_ = 0.42 e Å^−3^
Δρ_min_ = −0.24 e Å^−3^

### 

Data collection: *SMART* (Bruker, 2007 ▶); cell refinement: *SAINT-NT* (Bruker, 2007 ▶); data reduction: *SAINT-NT*; program(s) used to solve structure: *SHELXS97* (Sheldrick, 2008 ▶); program(s) used to refine structure: *SHELXL97* (Sheldrick, 2008 ▶); molecular graphics: *ORTEP-3 for Windows* (Farrugia, 2012 ▶); software used to prepare material for publication: *SHELXTL* (Sheldrick, 2008 ▶).

## Supplementary Material

Crystal structure: contains datablock(s) I, New_Global_Publ_Block. DOI: 10.1107/S1600536813027815/rn2119sup1.cif

Structure factors: contains datablock(s) I. DOI: 10.1107/S1600536813027815/rn2119Isup2.hkl

Supplementary material file. DOI: 10.1107/S1600536813027815/rn2119Isup3.cml

Additional supplementary materials:  crystallographic information; 3D view; checkCIF report

## Figures and Tables

**Table 1:** Hydrogen-bond geometry (Å, °)

*D*—H⋯*A*	*D*—H	H⋯*A*	*D*⋯*A*	*D*—H⋯*A*
O1—H1⋯N1	0.82	2.19	2.657 (3)	117
O1—H1⋯O2^i^	0.82	2.53	3.180 (5)	137
C10—H10⋯O1^ii^	0.93	2.51	3.400 (3)	160
C11—H11⋯N1	0.93	2.53	2.857 (4)	101

Symmetry codes: (i) 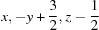; (ii) 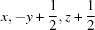.

**Fig. 1.**
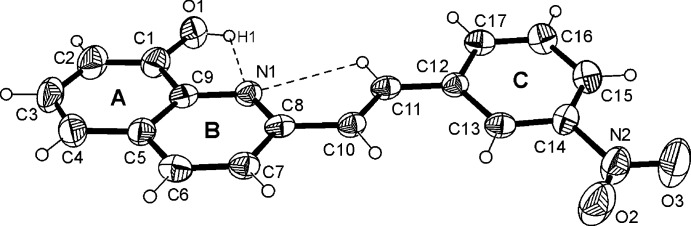
Perspective view of the title compound including atom labeling and ring specification. Thermal ellipsoids for the non-hydrogen atoms are drawn at the 50% probability level. Broken lines represent hydrogen bonds.

**Fig. 2.**
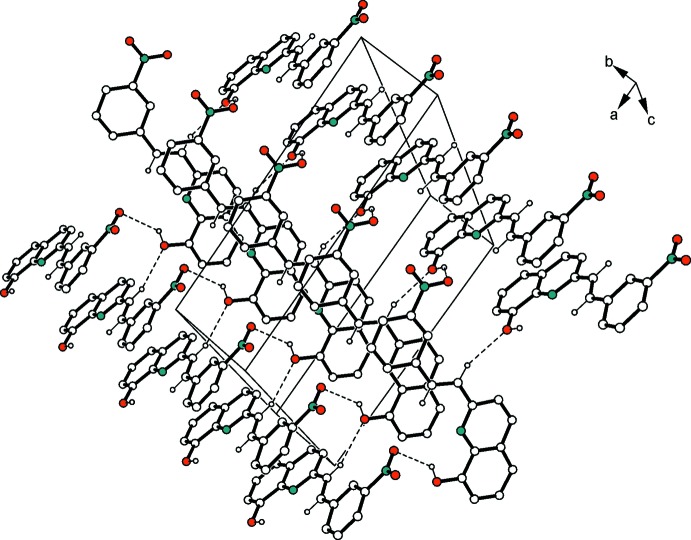
Packing structure of the title compound showing hydrogen bond interactions as broken lines.

## supplementary crystallographic information

### 1. Comment

Quinolin-8-ol (8-hydroxyquinoline) is a well known complexant for a variety of transition metal ions forming quantitatively precipitable internal complexes thus playing an important role in analytical chemistry (Albrecht *et al.*, 2008). Derivatives of quinolin-8-ol have also been used as an active component in the design of alkali and alkaline earth metal ion complexing ligands (Vögtle & Weber, 1979; Weber & Vögtle, 1975). Moreover, quinolin-8-ol shows strong fungicidal and antiseptic effects (Desvignes & Leguen, 1963) and derivatives of which are drugs for the successful treatment of different diseases (Cacciatore *et al.*, 2013; McMaster & Bruner, 1935). On the other hand, stilbene and its derivatives are starting materials for the preparation of various dyes, optical brighteners, liquid crystalline compounds and synthetic estrogens (Navadiya *et al.*, 2008; Ravikrishnan *et al.*, 2012; Waibel *et al.*, 2009; Zhu *et al.*, 2013). They are also of interest due to their particular stereochemistry including photochemical rearrangement and reactions (Butkovic *et al.*, 2011; Ho *et al.*, 2000). The structure of the title compound comprises both these specific construction elements, quinolin-8-ol and stilbene. In the structure of the compound (Fig. 1), the dihedral angle formed by the least-squares planes of the phenyl ring and the quinoline moiety is 11.0 (1)°, while the nitro substituent is twisted at an angle of 7.9 (2)° with reference to the phenyl ring. The bond lengths within the quinoline fragment are in the range of expected values and agree well with those found in the crystal structures of related compounds (Yoneda *et al.*, 2002; Zeng *et al.*, 2007). The torsion angle along the atomic sequence N(1)—C(8)—C(10)—C(11) is 6.3 (4)°. Within the quinolin-8-ol part, the hydroxyl hydrogen is connected to the nitrogen by a strained hydrogen bond [O(1)—H(1)···N(1) 2.19Å, 117°] which is typical for this kind of compounds (Fazaeli *et al.*, 2008; Malecki *et al.*, 2010; Zeng *et al.*, 2007). Moreover, there is a hydrogen bond type contact between H(11) and N(1) [*d*(H···N) 2.53Å]. The crystal (Fig. 2) is constructed of molecular stacks extending along the *b*-axis. The closest centroid-centroid distance between the phenol and pyridine ring of consecutive molecules is 4.02Å thus indicating, however, no π-stacking interaction between those groups. Moreover, the ethenylene fragment of a given molecule is sandwiched in a distance of *ca*. 3.45Å between the electron deficient aromatic rings of adjacent molecules. Interstack association is accomplished by a close network of O—H···O [*d*(O···O) 3.180 (5)Å] and C—H···O hydrogen bonds [*d*(C···O) 3.400 (3)Å] (Desiraju & Steiner, 1999).

### 2. Experimental

The title compound was synthesized *via* Knoevenagel type condensation (Yuan *et al.*, 2012) using 8-hydroxyquinaldine (320 mg, 2.0 mmol) and 4-nitrobenzaldehyde (1.21 g, 8.0 mmol) in acetic anhydride (100 ml). The mixture was stirred for 30 h under reflux. After removal of the solvent, the residue was dissolved in 100 ml of pyridine/water (*v*/*v* = 4:1) and heated at 100° for 1 h. Evaporation of the solvent under vacuum and purification of the crude product by recrystallization from ethanol yielded 230 mg (40%) of yellow crystals determined as the *E* configurated compound by ^1^H NMR analysis (ethenylene protons); m. p. = 449 K. IR (KBr) 3410, 3081, 1526, 1348, 1238, 1196, 829, 735, 593. ^1^H NMR (500 MHz, CDCl_3_) 7.20 (d, ^3^J_HH_ = 7.5 Hz, 1 H), 7.33 (d, ^3^J_HH_ = 8.2 Hz, 1 H), 7.51 - 7.39 (m, 2 H), 7.59 (t, ^3^J_HH_ = 7.9 Hz, 1 H), 7.65 (d, ^3^J_HH_ = 8.5 Hz, 1 H), 7.77 (d, ^3^J_HH_ = 16.1 Hz, 1 H), 7.92 (d, ^3^J_HH_ = 7.7 Hz, 1 H), 8.17 (^3^J_HH_ = 8.0 Hz, 1 H), 8.19 (^3^J_HH_ = 4.7 Hz, 1 H), 8.49 (s, 1 H). ^13^C NMR (126 MHz, CDCl_3_) 110.4, 117.7, 120.61, 121.6, 123.04, 127.8, 127.9, 129.8, 131.0, 131.5, 132.9, 136.8, 138.1, 138.3, 148.8, 152.2, 152.5. MS (ESI) *m/z*: found 293.0 [*M*+H]+; calc. for C_17_H_12_N_2_O_3_ 292.08. The melting point (uncorrected) was measured on a hot stage microscope (Büchi 510). The IR spectrum was recorded on a Perkin Elmer FT–IR 1600 spectrometer, ^1^H and ^13^C NMR spectra were measured on a Bruker Avance AV-500 spectrometer using (CH_3_)_4_Si as internal standard. The ESI mass spectrum was obtained using a ThermoFisher Scientific Orbitrap XL spectrometer. Crystals of the title compound suitable for X-ray structural analysis were taken from the crystallized product.

### 3. Refinement

H atoms were positioned geometrically and allowed to ride on their respective parent atoms, with C—H = 0.93 Å and *U*_iso_(H) = 1.2 *U*_eq_(C) and O—H = 0.82 Å and *U*_iso_(H) = 1.5 *U*_eq_(O).

### Crystal data

**Table:**

C_17_H_12_N_2_O_3_	*F*(000) = 608
*M**_r_* = 292.29	*D*_x_ = 1.377 Mg m^−^^3^
Monoclinic, *P*2_1_/*c*	Mo *K*α radiation, λ = 0.71073 Å
*a* = 20.3346 (7) Å	Cell parameters from 6524 reflections
*b* = 4.7167 (1) Å	θ = 2.6–24.8°
*c* = 15.5674 (6) Å	µ = 0.10 mm^−^^1^
β = 109.255 (2)°	*T* = 298 K
*V* = 1409.58 (8) Å^3^	Plate, colourless
*Z* = 4	0.54 × 0.24 × 0.06 mm

### Data collection

**Table:**

Bruker X8 APEXII CCD detector diffractometer	2655 independent reflections
Radiation source: fine-focus sealed tube	1768 reflections with *I* > 2σ(*I*)
Graphite monochromator	*R*_int_ = 0.028
phi and ω scans	θ_max_ = 25.6°, θ_min_ = 2.8°
Absorption correction: multi-scan (*SADABS*; Bruker, 2007)	*h* = −24→24
*T*_min_ = 0.950, *T*_max_ = 0.994	*k* = −4→5
22418 measured reflections	*l* = −18→18

### Refinement

**Table:**

Refinement on *F*^2^	Primary atom site location: structure-invariant direct methods
Least-squares matrix: full	Secondary atom site location: difference Fourier map
*R*[*F*^2^ > 2σ(*F*^2^)] = 0.065	Hydrogen site location: inferred from neighbouring sites
*wR*(*F*^2^) = 0.226	H-atom parameters constrained
*S* = 1.03	*w* = 1/[σ^2^(*F*_o_^2^) + (0.1192*P*)^2^ + 0.8405*P*] where *P* = (*F*_o_^2^ + 2*F*_c_^2^)/3
2655 reflections	(Δ/σ)_max_ < 0.001
200 parameters	Δρ_max_ = 0.42 e Å^−^^3^
0 restraints	Δρ_min_ = −0.24 e Å^−^^3^

### Special details

**Table:**

Geometry. All e.s.d.'s (except the e.s.d. in the dihedral angle between two l.s. planes) are estimated using the full covariance matrix. The cell e.s.d.'s are taken into account individually in the estimation of e.s.d.'s in distances, angles and torsion angles; correlations between e.s.d.'s in cell parameters are only used when they are defined by crystal symmetry. An approximate (isotropic) treatment of cell e.s.d.'s is used for estimating e.s.d.'s involving l.s. planes.
Refinement. Refinement of *F*^2^ against ALL reflections. The weighted *R*-factor *wR* and goodness of fit *S* are based on *F*^2^, conventional *R*-factors *R* are based on *F*, with *F* set to zero for negative *F*^2^. The threshold expression of *F*^2^ > σ(*F*^2^) is used only for calculating *R*-factors(gt) *etc*. and is not relevant to the choice of reflections for refinement. *R*-factors based on *F*^2^ are statistically about twice as large as those based on *F*, and *R*- factors based on ALL data will be even larger.

### Fractional atomic coordinates and isotropic or equivalent isotropic displacement parameters (Å^2^)

**Table:**

	*x*	*y*	*z*	*U*_iso_*/*U*_eq_	
O1	0.27846 (16)	0.0783 (7)	0.15082 (15)	0.1029 (10)	
H1	0.2582	0.1861	0.1750	0.154*	
O2	0.14581 (19)	1.0322 (8)	0.62596 (19)	0.1228 (12)	
O3	0.0587 (2)	1.2936 (9)	0.5668 (2)	0.1474 (16)	
N2	0.10391 (17)	1.1402 (7)	0.5616 (2)	0.0830 (9)	
C1	0.33187 (19)	−0.0492 (8)	0.2157 (2)	0.0714 (9)	
C2	0.3740 (2)	−0.2379 (8)	0.1950 (3)	0.0846 (11)	
H2	0.3665	−0.2812	0.1342	0.101*	
C3	0.4278 (2)	−0.3683 (8)	0.2610 (3)	0.0865 (11)	
H3	0.4555	−0.4993	0.2444	0.104*	
C4	0.44097 (17)	−0.3063 (7)	0.3524 (3)	0.0744 (9)	
H4	0.4776	−0.3938	0.3969	0.089*	
C5	0.39863 (14)	−0.1111 (6)	0.3769 (2)	0.0566 (7)	
C6	0.40520 (16)	−0.0303 (6)	0.4665 (2)	0.0616 (8)	
H6	0.4400	−0.1100	0.5153	0.074*	
C7	0.36152 (15)	0.1611 (6)	0.48187 (18)	0.0565 (7)	
H7	0.3666	0.2139	0.5413	0.068*	
C8	0.30746 (14)	0.2841 (6)	0.40839 (16)	0.0489 (7)	
N1	0.29937 (12)	0.2128 (5)	0.32357 (14)	0.0517 (6)	
C9	0.34298 (14)	0.0229 (6)	0.30808 (17)	0.0517 (7)	
C10	0.26019 (14)	0.4869 (6)	0.42662 (17)	0.0501 (7)	
H10	0.2641	0.5191	0.4870	0.060*	
C11	0.21158 (14)	0.6305 (6)	0.36290 (17)	0.0519 (7)	
H11	0.2087	0.5966	0.3029	0.062*	
C12	0.16274 (13)	0.8338 (6)	0.37686 (17)	0.0488 (6)	
C13	0.15702 (14)	0.8923 (6)	0.46277 (17)	0.0547 (7)	
H13	0.1858	0.8007	0.5144	0.066*	
C14	0.10879 (15)	1.0851 (7)	0.4697 (2)	0.0602 (7)	
C15	0.06455 (16)	1.2257 (7)	0.3969 (2)	0.0671 (8)	
H15	0.0317	1.3532	0.4036	0.081*	
C16	0.07075 (16)	1.1703 (7)	0.3126 (2)	0.0664 (8)	
H16	0.0421	1.2653	0.2617	0.080*	
C17	0.11795 (15)	0.9795 (6)	0.30281 (18)	0.0577 (7)	
H17	0.1204	0.9455	0.2451	0.069*	

### Atomic displacement parameters (Å^2^)

**Table:**

	*U*^11^	*U*^22^	*U*^33^	*U*^12^	*U*^13^	*U*^23^
O1	0.130 (2)	0.125 (2)	0.0489 (13)	0.0300 (19)	0.0224 (14)	−0.0120 (14)
O2	0.145 (3)	0.165 (3)	0.0645 (16)	0.054 (2)	0.0430 (18)	0.0067 (18)
O3	0.154 (3)	0.200 (4)	0.101 (2)	0.091 (3)	0.058 (2)	−0.015 (2)
N2	0.088 (2)	0.100 (2)	0.0637 (18)	0.0185 (19)	0.0287 (16)	−0.0100 (17)
C1	0.086 (2)	0.076 (2)	0.0586 (18)	−0.0004 (18)	0.0330 (17)	−0.0096 (16)
C2	0.109 (3)	0.080 (2)	0.080 (2)	0.000 (2)	0.051 (2)	−0.0205 (19)
C3	0.094 (3)	0.061 (2)	0.128 (3)	−0.0008 (19)	0.068 (3)	−0.019 (2)
C4	0.0660 (19)	0.0581 (18)	0.105 (3)	0.0024 (15)	0.0357 (18)	0.0008 (18)
C5	0.0559 (15)	0.0470 (15)	0.0735 (19)	−0.0086 (13)	0.0305 (14)	−0.0034 (13)
C6	0.0623 (17)	0.0632 (17)	0.0550 (16)	−0.0050 (15)	0.0138 (14)	0.0103 (14)
C7	0.0643 (17)	0.0605 (16)	0.0432 (14)	−0.0020 (14)	0.0157 (12)	0.0030 (13)
C8	0.0577 (15)	0.0509 (14)	0.0379 (13)	−0.0130 (12)	0.0155 (11)	0.0006 (11)
N1	0.0581 (13)	0.0520 (12)	0.0461 (12)	−0.0026 (11)	0.0187 (10)	−0.0005 (10)
C9	0.0590 (16)	0.0519 (15)	0.0479 (14)	−0.0108 (13)	0.0226 (13)	−0.0018 (12)
C10	0.0565 (15)	0.0559 (15)	0.0389 (13)	−0.0047 (12)	0.0170 (12)	−0.0016 (11)
C11	0.0623 (15)	0.0545 (15)	0.0370 (12)	−0.0124 (13)	0.0139 (12)	−0.0015 (12)
C12	0.0520 (14)	0.0475 (14)	0.0474 (14)	−0.0085 (12)	0.0174 (11)	−0.0020 (11)
C13	0.0598 (16)	0.0580 (16)	0.0425 (14)	−0.0071 (13)	0.0117 (12)	0.0031 (12)
C14	0.0607 (16)	0.0646 (17)	0.0587 (17)	0.0003 (14)	0.0242 (14)	−0.0087 (14)
C15	0.0585 (17)	0.0667 (19)	0.071 (2)	0.0029 (15)	0.0138 (15)	−0.0071 (16)
C16	0.0622 (17)	0.0694 (19)	0.0592 (18)	0.0031 (16)	0.0085 (14)	−0.0013 (15)
C17	0.0622 (16)	0.0618 (17)	0.0430 (14)	−0.0091 (14)	0.0094 (13)	0.0013 (13)

### Geometric parameters (Å, º)

**Table:**

O1—C1	1.356 (4)	C7—H7	0.9300
O1—H1	0.8200	C8—N1	1.319 (3)
O2—N2	1.195 (4)	C8—C10	1.449 (4)
O3—N2	1.194 (4)	N1—C9	1.337 (3)
N2—C14	1.488 (4)	C10—C11	1.332 (4)
C1—C2	1.346 (5)	C10—H10	0.9300
C1—C9	1.421 (4)	C11—C12	1.447 (4)
C2—C3	1.375 (6)	C11—H11	0.9300
C2—H2	0.9300	C12—C17	1.392 (4)
C3—C4	1.390 (5)	C12—C13	1.408 (4)
C3—H3	0.9300	C13—C14	1.368 (4)
C4—C5	1.396 (4)	C13—H13	0.9300
C4—H4	0.9300	C14—C15	1.365 (4)
C5—C6	1.410 (4)	C15—C16	1.384 (4)
C5—C9	1.424 (4)	C15—H15	0.9300
C6—C7	1.342 (4)	C16—C17	1.361 (4)
C6—H6	0.9300	C16—H16	0.9300
C7—C8	1.423 (4)	C17—H17	0.9300
			
C1—O1—H1	109.5	C8—N1—C9	118.7 (2)
O3—N2—O2	123.6 (3)	N1—C9—C1	116.7 (3)
O3—N2—C14	117.9 (3)	N1—C9—C5	124.8 (2)
O2—N2—C14	118.5 (3)	C1—C9—C5	118.5 (3)
C2—C1—O1	122.1 (3)	C11—C10—C8	124.6 (2)
C2—C1—C9	120.0 (3)	C11—C10—H10	117.7
O1—C1—C9	118.0 (3)	C8—C10—H10	117.7
C1—C2—C3	121.9 (3)	C10—C11—C12	127.1 (2)
C1—C2—H2	119.1	C10—C11—H11	116.5
C3—C2—H2	119.1	C12—C11—H11	116.5
C2—C3—C4	120.6 (3)	C17—C12—C13	117.0 (3)
C2—C3—H3	119.7	C17—C12—C11	119.8 (2)
C4—C3—H3	119.7	C13—C12—C11	123.3 (2)
C3—C4—C5	119.3 (3)	C14—C13—C12	119.5 (3)
C3—C4—H4	120.3	C14—C13—H13	120.3
C5—C4—H4	120.3	C12—C13—H13	120.3
C4—C5—C6	125.6 (3)	C15—C14—C13	123.4 (3)
C4—C5—C9	119.7 (3)	C15—C14—N2	118.6 (3)
C6—C5—C9	114.6 (3)	C13—C14—N2	118.0 (3)
C7—C6—C5	120.4 (3)	C14—C15—C16	117.0 (3)
C7—C6—H6	119.8	C14—C15—H15	121.5
C5—C6—H6	119.8	C16—C15—H15	121.5
C6—C7—C8	120.8 (3)	C17—C16—C15	121.3 (3)
C6—C7—H7	119.6	C17—C16—H16	119.3
C8—C7—H7	119.6	C15—C16—H16	119.3
N1—C8—C7	120.6 (3)	C16—C17—C12	121.8 (3)
N1—C8—C10	119.5 (2)	C16—C17—H17	119.1
C7—C8—C10	119.9 (2)	C12—C17—H17	119.1
			
O1—C1—C2—C3	−179.5 (4)	C4—C5—C9—C1	0.5 (4)
C9—C1—C2—C3	0.8 (6)	C6—C5—C9—C1	−179.1 (3)
C1—C2—C3—C4	−0.7 (6)	N1—C8—C10—C11	−6.3 (4)
C2—C3—C4—C5	0.5 (5)	C7—C8—C10—C11	174.4 (3)
C3—C4—C5—C6	179.2 (3)	C8—C10—C11—C12	179.6 (2)
C3—C4—C5—C9	−0.4 (4)	C10—C11—C12—C17	175.9 (3)
C4—C5—C6—C7	179.7 (3)	C10—C11—C12—C13	−4.9 (4)
C9—C5—C6—C7	−0.7 (4)	C17—C12—C13—C14	0.1 (4)
C5—C6—C7—C8	0.6 (4)	C11—C12—C13—C14	−179.2 (2)
C6—C7—C8—N1	−0.2 (4)	C12—C13—C14—C15	0.5 (4)
C6—C7—C8—C10	179.0 (2)	C12—C13—C14—N2	179.6 (3)
C7—C8—N1—C9	0.0 (4)	O3—N2—C14—C15	4.2 (5)
C10—C8—N1—C9	−179.2 (2)	O2—N2—C14—C15	−174.8 (4)
C8—N1—C9—C1	179.4 (2)	O3—N2—C14—C13	−175.0 (4)
C8—N1—C9—C5	−0.1 (4)	O2—N2—C14—C13	6.0 (5)
C2—C1—C9—N1	179.6 (3)	C13—C14—C15—C16	−1.2 (5)
O1—C1—C9—N1	0.0 (4)	N2—C14—C15—C16	179.7 (3)
C2—C1—C9—C5	−0.7 (5)	C14—C15—C16—C17	1.3 (5)
O1—C1—C9—C5	179.6 (3)	C15—C16—C17—C12	−0.8 (5)
C4—C5—C9—N1	−179.9 (3)	C13—C12—C17—C16	0.1 (4)
C6—C5—C9—N1	0.5 (4)	C11—C12—C17—C16	179.3 (3)

### Hydrogen-bond geometry (Å, º)

**Table:**

*D*—H···*A*	*D*—H	H···*A*	*D*···*A*	*D*—H···*A*
O1—H1···N1	0.82	2.19	2.657 (3)	117
O1—H1···O2^i^	0.82	2.53	3.180 (5)	137
C10—H10···O1^ii^	0.93	2.51	3.400 (3)	160
C11—H11···N1	0.93	2.53	2.857 (4)	101

Symmetry codes: (i) *x*, −*y*+3/2, *z*−1/2; (ii) *x*, −*y*+1/2, *z*+1/2.
